# Data on prolonged morphine-induced antinociception and behavioral inhibition in older rats

**DOI:** 10.1016/j.dib.2018.05.001

**Published:** 2018-05-09

**Authors:** Alok Kumar Paul, Nuri Gueven, Nikolas Dietis

**Affiliations:** Division of Pharmacy, School of Medicine, University of Tasmania, Australia

## Abstract

This article contains supportive data related to a research article entitled “Age-dependent antinociception and behavioral inhibition by morphine” (Paul et al., 2018) [1]. Antinociceptive latencies of 8 and 24-week old rats were obtained from tail-flick and hot plate assays after morphine treatment. Motor behavioral effects were measured at different time-points using automated infrared tracking in an open-field arena. Residual morphine content in post-mortem tissues were measured 240 min post-treatment. Concurrent measurements of antinociception, motor behavior and residual morphine content in post-mortem tissues of 8-week and 24-week old morphine-treated rats provide an integrated assessment of age-related differences.

**Specifications Table**

**Table:** 

Subject area	*Biology, psychology, drug response*
More specific subject area	*Motor behavior, antinociception, age*
Type of data	*Table, text file, figure*
How data was acquired	*Tail-flick apparatus, Hot-plate apparatus, TSE MCS box (open-field paradigm), ELISA*
Data format	*Analyzed*
Experimental factors	*Age*
Experimental features	*Male Sprague-Dawley rats were tested for antinociceptive and open-field motor behavior before and at different time-points after morphine-treatment.*
Data source location	*Division of Pharmacy, School of Medicine, University of Tasmania, Hobart, TAS, Australia*
Data accessibility	*Data presented in this brief is related to a study published in parallel*[1]

**Value of the data**

• Data presented highlights differential age-dependent antinociceptive effects of morphine.
• The results illustrate age-related differential effects of morphine on motor behavior.
• The data connect antinociception, motor behavior and residual morphine tissue levels as a function of animal age.

## 1 Data

**Fig. 1:**
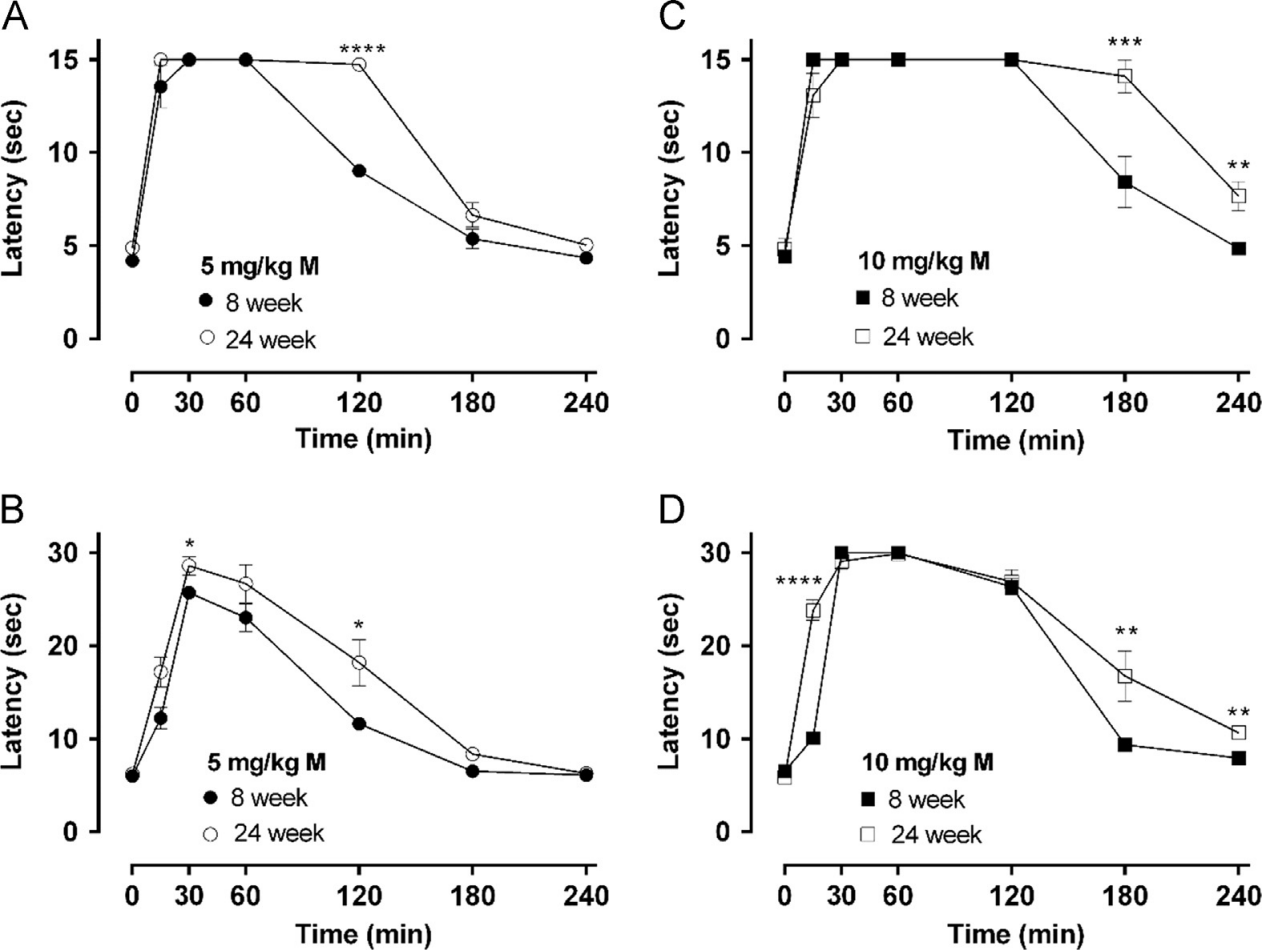
Antinociceptive latencies of morphine in 8 and 24 week old rats. Antinociceptive profiles of 8 and 24 week old rats treated with subcutaneous morphine (5 and 10 mg/kg) pre- and post-administration (15–240 min) was measured using a tail-flick (A, C) and hot-plate assay (B, D). Antinociceptive curves of morphine 5 mg/kg (A, B) and 10 mg/kg (C, D) are presented as latency (in sec) against pre- and post-administration time-points as described in Section 2. All data are presented as mean±SEM, *n*=5 per group. Statistically significant differences compared against 8-week old animals for the same time-point were generated using repeated measure one-way ANOVA with Sidak's multiple comparisons test (^*^p<0.05, ^**^p<0.01, ^***^p<0.001 and ^****^p<0.0001).

**Fig. 2:**
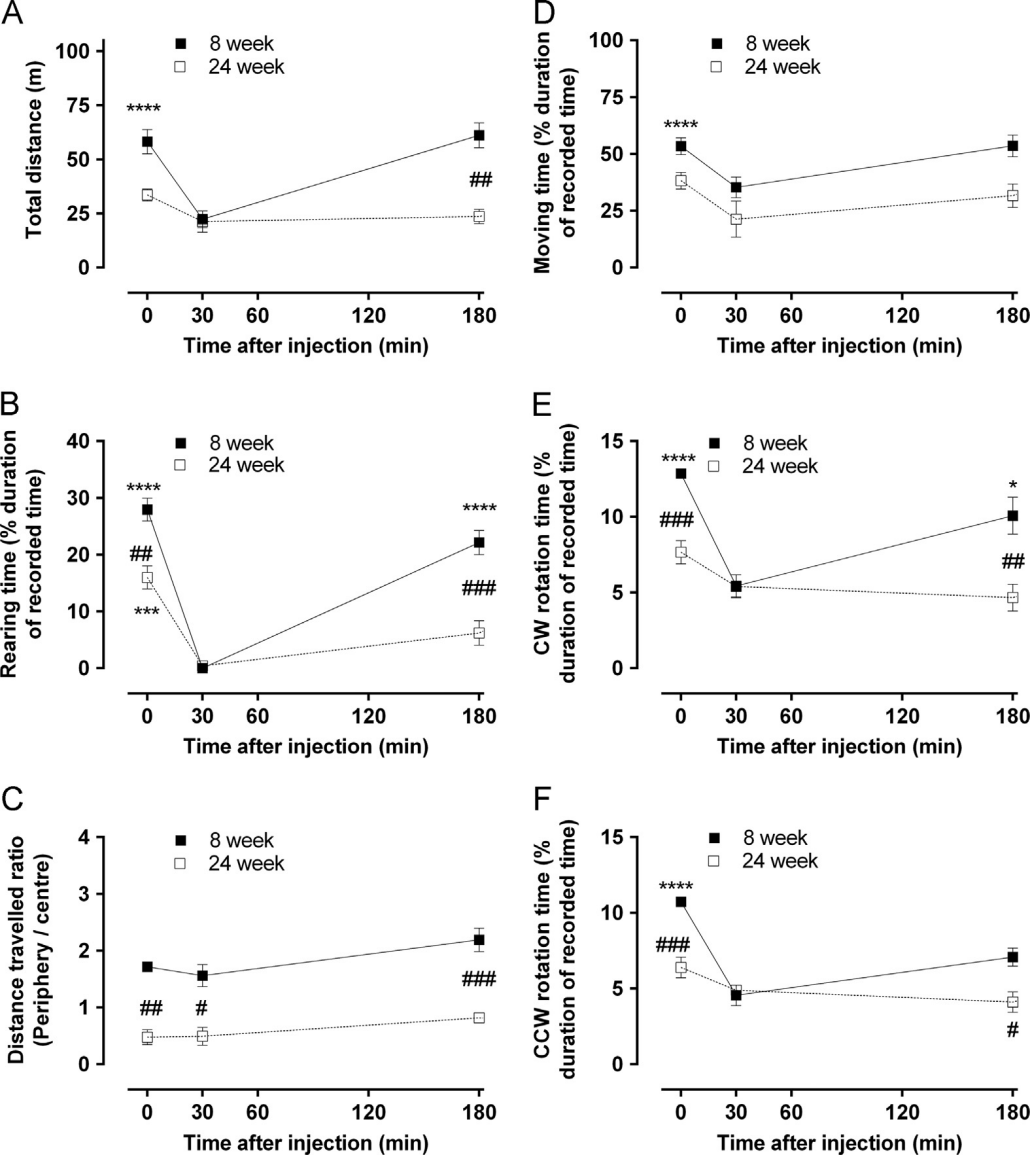
Detailed analysis of high dose morphine-induced behavioral changes. Six distinct animal behavior (A. Moving time, B. distance, C. rearing time, D. clockwise (CW) rotation time, E. Counter-clockwise (CCW) rotation time, and F. the ratio of distance travelled in periphery vs center were recorded after a single-dose of morphine (10 mg/kg, s.c.) in 8 and 24 week old rats and were analyzed using ActiMot software (TSE Systems). Measurements were taken prior to drug administration (basal), 30 and 180 min post-administration, as described in Section 2. Differences between the 30 min time point and the basal or 180 min time points were significantly different (*p<0.05, ***p<0.001 and ****p<0.0001, using repeated measure one-way ANOVA with Sidak's multiple comparison *post hoc* test). Likewise, significant differences against 8 week old animals were observed for some time points (^##^*p*<0.01 and ^###^*p*<0.001, using non-repeated measure one-way ANOVA with Sidak's multiple comparison *post hoc* test). All data are presented as mean±SEM, *n*=5 per group.

**Fig. 3:**
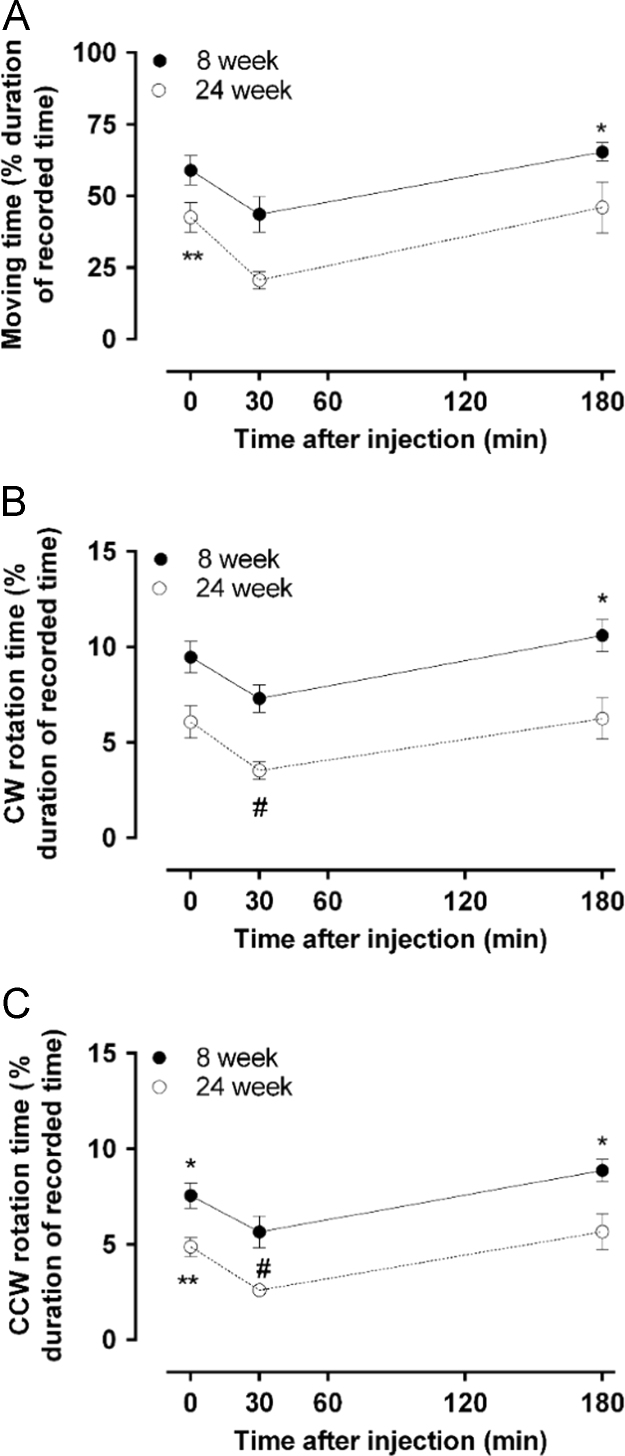
Detailed analysis of low-dose morphine-induced behavioral changes. Three distinct animal behavior (A. Moving time, B. Clockwise (CW) rotation time, C. Counter-clockwise (CCW) rotation time) were recorded after a single-dose of morphine (5 mg/kg, s.c.) in 8 and 24 week old rats and analyzed using ActiMot software (TSE Systems). Measurements were taken prior to drug administration (basal), 30 and 180 min post-administration, as described in Section 2. Differences between the 30 min time point and the basal or 180 min time points were significantly different (*p<0.05, ***p<0.001 and ****p<0.0001, using repeated measure one-way ANOVA with Sidak's multiple comparison *post hoc* test). Likewise, significant differences against 8 week old animals were observed for some time points (^##^*p*<0.01 and ^###^*p*<0.001, using non-repeated measure one-way ANOVA with Sidak's multiple comparison *post hoc* test). All data are presented as mean±SEM, *n*=5 per group.

**Fig. 4:**
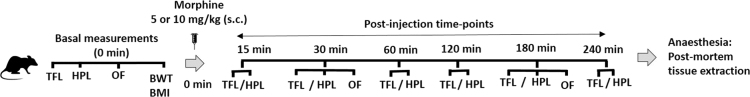
Schematic diagram of the morphine treatment and assessment protocol. TFL: tail-flick latency; HPL: hot-plate latency, OF: open-field test; BWT: body-weight; BMI: body mass index; 0 min: base line before treatment.

The data presented in this article provide information on the differences in antinociceptive latencies and motor behavior between 8 and 24-week old rats after a single subcutaneous (s.c.) injection of 5 or 10 mg/kg morphine. Fig. 1 shows antinociceptive latencies of 8 and 24-week old rats. The corresponding antinociceptive effects (shown as maximum possible effect, MPE %) were reported previously [1]. Fig. 2 describes six different motor behaviors of 8 and 24-week old rats after 10 mg/kg morphine injection. Fig. 3 presents three different motor behaviors of rats treated with 5 mg/kg morphine. The data regarding distance travelled, rearing and the ratio of distance travelled in the periphery vs center for 5 mg/kg morphine treated rats was described previously [1]. After antinociception testing at 240 min post-injection of 5 mg/kg morphine, the residual amount of morphine present in different post-mortem tissues was determined and compared between groups using non-repeated measure two-way ANOVA (Table 1). Table 1 shows the data that was previously represented graphically as Fig. 4A and B [1].

**Table 1:** Residual morphine content in post-mortem tissues after 4 h of injections of 5 mg/kg morphine.

**Residual morphine (μg) (±SEM)**						
**Tissue**	***8 week***	***24 week***	***p value***	***8 week***	***24 week***	***p value***
	***per tissue weight*****(g)**	***per tissue weight*****(g)**		***per protein content*****(g)**	***per protein content*****(g)**	
**Serum**	4.47±1.72	23.32±1.61	>0.05	0.49±0.12	3.39±0.34	<0.0001
**Spleen**	22.45±2.93	68.41±7.05	<0.001	0.89±0.14	4.63±0.35	<0.0001
**Kidney**	58.66±5.65	56.14±5.95	>0.05	2.42±0.53	4.23±0.15	<0.05
**Liver**	40.12±6.46	44.52±1.01	>0.05	3.25±0.37	2.81±0.71	>0.05
**Heart**	1.62±0.31	20.49±2.31	<0.0001	0.43±0.31	1.20±0.62	>0.05
**Lungs**	17.38±3.61	63.22±14.8	<0.001	0.96±0.32	2.28±0.54	>0.05
**Brain**	1.21±0.49	7.47±0.82	<0.05	0.25±0.11	0.41±0.18	>0.05

## 2 Experimental design, materials and methods

### 2.1 Animals

Animal handling procedures were performed according to the University of Tasmania Animal Ethics Committee (approval no. A00013864) and The Australian Code for the Care and Use of Animals for Scientific Purposes [2]. 8-week (*n*=10) and 24-week old (*n*=10) male Sprague Dawley (SD) rats were used in this study which were raised on normal diet. During the experiments, all animals were single-housed under standard laboratory conditions. The 8 week and 24 week old rats were further divided randomly into two subgroups as previously described [3]. Each group received a different dose of morphine sulphate solution (5 mg/kg or 10 mg/kg) as single *s.c.* injection. These doses of morphine were chosen from one of our previous studies [4]. The body-weight of all animals was recorded prior to morphine administration of and used to calculate the actual amount of morphine per animal. Detailed procedures of animal handling and treatment were described previously [1].

### 2.2 Nociception testing

Antinociception was tested using tail-flick and hot-plate assays performed randomly with an interval of 1 min between the two assays, using specialized commercial instruments (Ugo Basile, Comerio, Italy). Maximum exposure of rats to the nociceptive thermal source (cut-off time) was 15 sec for the tail-flick and 30 sec for the hot-plate assay. The hot-plate temperature was set at 54±0.5 °C. All rats were tested immediately prior to morphine administration (basal latency) and at 15, 30, 60, 120, 180 and 240 min post-administration in both assays. Nociception testing was conducted in a blinded manner and the results were recorded by averaging three independent tests for each time-point with a 1 min time interval in between.

### 2.3 Behavioral testing

Motor behavior of the animals were measured using six different parameters (moving time, total distance travelled, rearing time, clockwise rotation, anti-clockwise rotation and topology (ratio of presence in periphery versus center)), as described previously [1]. The behavioral data was obtained using an open-field arena in a Multi-Conditioning System (MCS) (TSE GmbH, Homburg, Germany) 2 min after nociception measurements at 0 min (pre), 30 and 180 min post-injections of morphine over a period of 5 min. The open-field area was placed in a noise/light/temperature insulated system and infrared-beams were used to detect animal movement. The open-field arena was cleaned thoroughly between each animal and a background sound (20 db) was used to cover outside noise. A schematic diagram represents the overall treatment protocol, antinociception and behavioral measurements (Fig. 4).

### 2.4 Tissue collection and residual morphine in post-mortem tissues

The detailed procedure for collection of post-mortem tissues and determination of morphine and protein contents in those tissues were described previously [1].

### 2.5 Statistical analysis

The data are presented as graphs or table displaying mean±SEM (standard error of mean) from *n* measurements. Student's *t*-test, one-way ANOVA or two-way ANOVA with Sidak's multiple comparison *post hoc* test were used for statistical comparison as appropriate, using GraphPad Prism V6.01 software (GraphPad Software Inc., La Jolla, CA, USA). Significance was set at *p<*0.05.
