# Supplementary material for: Data on prolonged morphine-induced antinociception and behavioral inhibition in older rats
Source: Data Brief. 2018 May 9;19:183–8. doi: 10.1016/j.dib.2018.05.001 (PMC5992970; doi:10.1016/j.dib.2018.05.001)
Supplement: Supplementary file 1 — Supplementary material [file mmc1.pdf]

Manuscript No.: DIB-D-18-00623
Title: Prolonged morphine-induced antinociception and behavioral inhibition in older rats
Journal Title: Data in Brief

<u>Author declaration</u>

All authors have read and approved the manuscript. The experiments were conducted according to the approval of the University of Tasmania Animal Ethics Committee (approval no. A00013864) and the *Australian Code for the Care and Use of Animals for Scientific Purposes*.

Conflicts of Interest: The authors declare no conflicts of interest.

Funding: The project was funded by the Division of Pharmacy, School of Medicine, University of Tasmania, Australia.

Contributions of Authors: A.K.P. designed the study, completed the experiments, contributed to the analysis of data and preparation of manuscript. N.G. designed the study and contributed to the preparation of manuscript. N.D. designed the study and contributed to the analysis of data and preparation of manuscript.

Signed by,

Alok Paul

Alok K. Paul (on behalf of all authors)
Date: April 04, 2018
